# Supplementary material for: Guillain-Barré syndrome after the Zika epidemic in Colombia: A multicenter, matched case-control study
Source: PLoS Negl Trop Dis. 2025 Mar 5;19(3):e0012898. doi: 10.1371/journal.pntd.0012898 (PMC11922255; doi:10.1371/journal.pntd.0012898)
Supplement: S1 Table — (DOCX) [file pntd.0012898.s001.docx]

# **S1 Table. GBS Demographics and Presenting Symptoms^a^ of Matched and Unmatched GBS Cases.**

| **Characteristics** | **GBS cases matched**  **N=57 (%)** | **GBS cases unmatched**  **N=25 (%)** |
| --- | --- | --- |
| Age in years, Median (Range) | 52 (3–82) | 49 (4–94) |
| Age >15 years old | 50 (88) | 24 (96) |
| Sex Male | 38 (67) | 19 (76) |
| Upper respiratory infection or influenza | 19 (33) | 11 (44) |
| Diarrhea at onset | 17 (30) | 7 (28) |
| Urinary tract infection | 0 | 1 (4) |
| Vaccination | 0 | 0 |
| Asthenia | 28 (49) | 12 (48) |
| Fever | 20 (35) | 10 (40) |
| Preceding Diarrhea (within 4 weeks) no present at onset | 16 (28) | 8 (32) |
| Myalgias | 16 (28) | 9 (36) |
| Nausea and/or vomiting | 11 (19) | 2 (8) |
| Headache | 11 (19) | 13 (52) |
| Arthralgia | 7 (12) | 5 (20) |
| Rash | 3 (5) | 0 |
| Asymptomatic^b^ | 14 (25) | 4 (16) |
| **Presenting Neurological Signs and Symptoms** |  |  |
| Days from onset to neurological symptoms, Median (Range) | 5 (0–35) | 4 (0–16) |
| Motor dysfunction^c^ | 56 (98) | 25 (100) |
| Ascending paralysis pattern | 33 (58) | 19/25 (76) |
| Lower limb paralysis | 43 (75) | 20 (80) |
| Upper limb paralysis | 28 (49) | 12 (48) |
| Descending paralysis | 7 (12) | 2 (8) |
| Sensory symptoms^d^ | 31 (54) | 13 (52) |
| Allodynia or neuropathic pain | 7 (12) | 3 (12) |
| Facial palsy | 5 (9) | 3 (12) |
| Dysautonomia | 5 (9) | 0 |
| Urinary incontinence | 2 (4) | 0 |
| Vertigo | 1 (2) | 0 |
| **Neurological Characteristics** |  |  |
| MRC Sum Score, Median (Range) | 30 (0–60) | 32 (0–60) |
| GBS disability score |  | 24/25 |
| 0 – Healthy |  | 0 |
| 1 – Minor symptoms capable of running |  | 2 (8) |
| 2 – Walks 10 m or more without assistance, unable to run |  | 0 |
| 3 – Walks 10 m across an open space with help |  | 4 (16) |
| 4 – Bedridden or chairbound |  | 9 (38) |
| 5 – Assisted ventilation for at least part of the day |  | 9 (38) |
| 6 – Death |  | 0 |
| Modified Rankin Scale, n/N | 57/57 | 24/25 |
| 0 – No symptoms | 0 | 0 |
| 1 – No significant disability | 1 (2) | 2 (8) |
| 2 – Slight disability | 4 (7) | 0 |
| 3 – Moderate disability | 6 (10) | 1 (4) |
| 4 – Moderately severe disability | 18 (32) | 9 (38) |
| 5 – Severe disability | 28 (49) | 12 (50) |
| 6 – Death | 0 | 0 |
| ICU admission, n/N (%) | 34/50 (68) | 13/22 (59) |
| Required mechanical ventilation | 8 (14) | 8 (32) |
| **CSF features, n/N** | **48/57** | **22/25** |
| CSF WBC, Median (Range) | 0 (0–41) | 2 (0–100) |
| CSF protein, Median (Range) | 67 (19–283) | 49 (19–15,300) |
| **Neuroconduction & EMG studies, n/N** | **43/57** | **11/25** |
| Primary demyelinating | 18 (42) | 6 (55) |
| Primary axonal | 15 (35) | 0 |
| Normal | 1 (2) | 0 |
| Equivocal | 4 (9) | 1 (9) |
| Unexcitable | 2 (5) | 1 (9) |
| Other | 3 (7) | 3 (27) |
| **Treatment** | **57 (%)** | **25 (%)** |
| IVIG | 31 (54) | 13 (52) |
| Plasma exchange | 20 (35) | 7 (28) |
| Steroids | 2 (4) | 0 |
| Other | 1 (2) | 1 (4) |
| No treatment | 3 (5) | 4 (16) |
| **Biological Samples Characteristics** |  |  |
| Days between sampling and onset of neurological symptoms, Median (Range) | 10 (1–33) | 11 (0–59) |
| Days between sampling and initiation of treatment, Median (Range) | 2 (-1­–3) | 5 (-10–2) |
| Relation between sampling and initiation of treatment, n/N | 54/57 | 21/25 |
| Before | 2 (4) | 2 (10) |
| Same day | 6 (11) | 2 (10) |
| After | 31 (57) | 11 (52) |
| Unknown | 15 (28) | 6 (28) |

^a^ Symptoms of systemic illness immediately preceding or during the onset of neurological symptoms.

^b^ No evidence of systemic illness when neurological symptoms presented

^c^ Motor dysfunction is the presence of muscle weakness in the lower and/or upper limb or paralysis.

^d^ Sensory symptoms are the presence of hypoesthesia, anesthesia, and/or paresthesias.
