# Supplementary material for: Guillain-Barré syndrome after the Zika epidemic in Colombia: A multicenter, matched case-control study
Source: PLoS Negl Trop Dis. 2025 Mar 5;19(3):e0012898. doi: 10.1371/journal.pntd.0012898 (PMC11922255; doi:10.1371/journal.pntd.0012898)
Supplement: S2 Table — (DOCX) [file pntd.0012898.s002.docx]

# **S2 Table. Molecular and Immunological Testing and Infectious Disease Diagnosis in GBS Cases and Controls**

| **Test performed** | **GBS cases matched**  **%** | **GBS cases unmatched**  **%** | **Hospital control**  **%** | **House control**  **%** |
| --- | --- | --- | --- | --- |
| **Zika virus** |  |  |  |  |
| **RT–PCR in any fluid^a^, n/N** | **57/57** | **19/25** | **22/22** | **55/55** |
| Positive | 2 | 0 | 0 | 0 |
| Negative | 98 | 100 | 100 | 100 |
| **RT–PCR in serum, n/N** | **57/57** | **19/25** | **22/22** | **55/55** |
| Negative | 100 | 100 | 100 | 100 |
| **RT–PCR in CSF, n/N** | **20/57** | **5/25** | **NA** | **NA** |
| Negative | 100 | 100 | – | – |
| **RT–PCR in urine, n/N** | **43/57** | **13/25** | **15/22** | **39/55** |
| Positive | 2 | 0 | 0 | 0 |
| Negative | 98 | 100 | 100 | 100 |
| **Dengue virus** |  |  |  |  |
| **RT–PCR in serum, n/N** | **57/57** | **18/25** | **22/22** | **55/55** |
| Negative | 100 | 100 | 100 | 100 |
| **Anti–Flavivirus IgG in serum, n/N** | **56/57** | **20/25** | **22/22** | **53/55** |
| Positive | 32 | 30 | 45 | 45 |
| Negative | 68 | 70 | 55 | 55 |
| **Anti–Flavivirus IgM in serum, n/N** | **57/57** | **20/25** | **22/22** | **54/55** |
| Positive | 3 | 10 | 23 | 4 |
| Negative | 97 | 90 | 77 | 96 |
| **Anti–Flavivirus IgG in CSF, n/N** | **6/55** | **2/25** | **NA** | **NA** |
| Positive | 0 | 0 | – | – |
| Negative | 100 | 100 | – | – |
| **Anti–Flavivirus IgM in CSF, n/N** | **6/55** | **2 /25** | **NA** | **NA** |
| Positive | 0 | 0 | – | – |
| Negative | 100 | 100 | – | – |
| **Flaviviruses serological diagnosis, n/N** | **56/57** | **20/25** | **22/22** | **52/55** |
| Recent | 3 | 10 | 23 | 4 |
| Exposed | 29 | 25 | 23 | 42 |
| Negative | 68 | 65 | 54 | 54 |
| **Chikungunya virus** |  |  |  |  |
| **RT–PCR in serum, n/N** | **57/57** | **19/25** | **22/22** | **55/55** |
| Negative | 100 | 100 | 100 | 100 |
| **RT–PCR in CSF, n/N** | **18/55** | **4/25** | **NA** | **NA** |
| Negative | 100 | 100 | – | – |
| **RT–PCR in urine, n/N** | **41/57** | **10/25** | **14/22** | **38/55** |
| Negative | 100 | 100 | 100 | 100 |
| **Anti–CHIK IgG in serum, n/N** | **57/57** | **14/25** | **20/22** | **55/55** |
| Positive | 33 | 50 | 35 | 25 |
| Negative | 67 | 50 | 65 | 75 |
| **Anti–CHIK IgM in serum, n/N** | **56/57** | **14/25** | **13/22** | **54/55** |
| Positive | 7 | 7 | 4 | 2 |
| Negative | 93 | 93 | 96 | 98 |
| **Serological diagnosis, n/N** | **56/57** | **14/25** | **20/22** | **54/55** |
| Recent | 7 | 7 | 5 | 2 |
| Exposed | 27 | 50 | 35 | 26 |
| Negative | 66 | 43 | 60 | 72 |
| ***Campylobacter jejuni*** |  |  |  |  |
| **Anti–*C. jejuni* IgG in serum, n/N** | **57/57** | **16/25** | **21/22** | **52/55** |
| Positive | 70 | 63 | 33 | 42 |
| Negative | 30 | 37 | 67 | 58 |
| **Anti–*C. jejuni* IgM in serum, n/N** | **55/57** | **13/25** | **22/22** | **55/55** |
| Positive | 11 | 0 | 5 | 5 |
| Negative | 89 | 100 | 95 | 95 |
| **Anti–*C. jejuni* IgA in serum, n/N** | **54/57** | **18/25** | **19/22** | **55/55** |
| Positive | 35 | 11 | 21 | 25 |
| Negative | 65 | 89 | 79 | 75 |
| **Serological diagnosis, n/N** | **57/57** | **18/25** | **22/22** | **55/55** |
| Recent | 33 | 5 | 9 | 14 |
| Exposed | 41 | 56 | 36 | 42 |
| Negative | 26 | 39 | 55 | 44 |
| ***Mycoplasma pneumoniae*** |  |  |  |  |
| **Anti*–M. pneumoniae* IgM in serum, n/N** | **57/57** | **14/25** | **22/22** | **55/55** |
| Positive | 21 | 7 | 14 | 15 |
| Negative | 72 | 86 | 86 | 80 |
| Indeterminate | 7 | 7 | 0 | 5 |
| **Serological diagnosis, n/N** | **57/57** | **14/25** | **22/22** | **55/55** |
| Recent | 21 | 7 | 14 | 15 |
| Negative | 72 | 86 | 86 | 80 |
| Undetermined | 7 | 7 | 0 | 5 |
| **Cytomegalovirus** |  |  |  |  |
| **Anti–CMV IgM in serum, n/N** | **57/57** | **14/25** | **22/22** | **55/55** |
| Positive | 28 | 21 | 23 | 18 |
| Negative | 70 | 72 | 68 | 73 |
| Indetermined | 2 | 7 | 9 | 9 |
| **CMV avidity IgG in serum ^b^, n/N** | **19/19** | **3/3** | **7/7** | **15/15** |
| Positive | 84 | 100 | 100 | 100 |
| Indetermined | 16 | 0 | 0 | 0 |
| **Serological diagnosis, n/N** | **56/57** | **13/25** | **22/22** | **55/55** |
| Primoinfection | 0 | 0 | 0 | 0 |
| Reactivation | 29 | 23 | 32 | 27 |
| Negative | 71 | 77 | 68 | 73 |
| **Epstein–Barr virus** |  |  |  |  |
| **anti–VCA in serum, n/N** | **57/57** | **14/25** | **22/22** | **54/55** |
| Positive | 5 | 7 | 0 | 2 |
| Negative | 95 | 93 | 100 | 98 |
| **IgG EBNA–1 in serum ^c^, n/N** | **3/3** | **1/1** | **NA** | **1/1** |
| Positive | 67 | 100 | – | 100 |
| Negative | 33 | 0 | – | 0 |
| **Serological diagnosis, n/N** | **57/57** | **14/25** | **22/22** | **55/55** |
| Primoinfection | 2 | 0 | 0 | 0 |
| Reactivation | 4 | 7 | 0 | 2 |
| Negative | 94 | 93 | 100 | 98 |
| **Varicella Zoster virus** |  |  |  |  |
| **IgM Serum, n/N** | **55/57** | **14/25** | **22/22** | **52/55** |
| Positive | 9 | 14 | 0 | 4 |
| Negative | 91 | 72 | 100 | 96 |
| Undetermined | 0 | 14 | 0 | 0 |
| **IgG avidity test Serum^d^, n/N** | **7/9** | **3/14** | **NA** | **5/5** |
| Positive | 100 | 100 | – | 100 |
| Indetermined | 0 | 0 | – | 0 |
| **Serological diagnosis, n/N** | **57/57** | **13/25** | **22/22** | **52/55** |
| Primoinfection | 0 | 0 | 0 | 0 |
| Reactivation | 12 | 23 | 0 | 9 |
| Negative | 88 | 77 | 100 | 91 |
| **Hepatitis E virus** |  |  |  |  |
| **IgM in serum, n/N** | **56/55** | **4/25** | **20/22** | **53/55** |
| Positive | 5 | 0 | 0 | 0 |
| Negative | 95 | 100 | 100 | 100 |
| **Summary of infections** | **N=57** | **N=25** | **N=22** | **N=55** |
| No infection | 18 | 44 | 32 | 29 |
| 1 infection | 49 | 44 | 32 | 42 |
| 2 or more infections | 33 | 12 | 36 | 29 |

n/N indicates the number of samples processed of the total to included.

^a^ Testing for Zika virus included at least blood, urine and/or CSF RT–PCR.

^b^ Testing of CMV IgG and IgG avidity test was done only when IgM was positive or equivocal to determine primoinfection (IgG avidity test negative while IgM positive) or reactivation (both IgG avidity and IgM tests positive).

^c^ Testing of EBV primoinfection of reactivation was assessed only in cases with positive or equivocal anti–EBV VCA IgM antibodies using anti–EBNA IgG testing. Cases positive anti–EBV VCA IgM and negative anti–EBNA IgG were considered primoinfection while both positive tests were considered reactivation.

^d^ Testing of VZV infection used IgG avidity assay to determine primoinfection. If the IgG OD value reduction was below 40% after urea treatment, it was considered a primary infection, if it was high (IgG OD value above 60% after urea treatment), it was considered as reactivation.
