# Supplementary material for: Guillain-Barré syndrome after the Zika epidemic in Colombia: A multicenter, matched case-control study
Source: PLoS Negl Trop Dis. 2025 Mar 5;19(3):e0012898. doi: 10.1371/journal.pntd.0012898 (PMC11922255; doi:10.1371/journal.pntd.0012898)
Supplement: S3 Table — (DOCX) [file pntd.0012898.s003.docx]

# **S3 Table. Microbiological Tests Results by Age Group in GBS cases**

| **Demographics and symptoms** | **Age group** | | |
| --- | --- | --- | --- |
|  | **< 15 years old** | **15 – 64 years old** | **≥ 65 years old** |
| **Zika virus** |  |  |  |
| RT–PCR in any fluid positive, n/N | 0/7 | 1/37 | 0/13 |
| RT–PCR in serum positive, n/N | 0/7 | 0/37 | 0/13 |
| RT–PCR in urine positive, n/N | 0/5 | 1/28 | 0/10 |
| RT–PCR in CSF positive, n/N | ND | 0/15 | 0/5 |
| **Dengue virus** |  |  |  |
| RT–PCR in serum positive, n/N | 0/7 | 0/37 | 0/13 |
| Anti–Flavivirus IgM in serum positive, n/N | 1/7 | 0/37 | 1/13 |
| Anti–Flavivirus IgG in serum positive, n/N | 1/7 | 11/36 | 6/13 |
| Flaviviruses serological, n/N |  |  |  |
| Recent | 1/7 | 0/36 | 1/13 |
| Exposed | 0/7 | 11/36 | 5/13 |
| Negative | 6/7 | 25/36 | 7/13 |
| **Chikungunya virus** |  |  |  |
| RT–PCR in serum positive, n/N | 0/7 | 0/37 | 0/13 |
| RT–PCR in CSF positive, n/N | ND | 0/13 | 0/5 |
| CHIK RT–PCR in urine positive, n/N | 0/4 | 0/27 | 0/10 |
| Anti–CHIK IgM in serum positive, n/N | 0/7 | 4/36 | 0/13 |
| Anti–CHIK IgG in serum positive, n/N | 0/7 | 18/37 | 1/13 |
| Serological diagnosis, n/N |  |  |  |
| Recent | 0/7 | 4/36 | 0/13 |
| Exposed | 0/7 | 14/36 | 1/13 |
| Negative | 7/7 | 18/36 | 12/13 |
| ***Campylobacter jejuni*** |  |  |  |
| Anti–*C. jejuni* IgM in serum positive, n/N | 3/7 | 3/36 | 1/12 |
| Anti–*C. jejuni* IgG in serum positive, n/N | 5/7 | 27/37 | 8/13 |
| Anti–*C. jejuni* IgA in serum positive, n/N | 3/7 | 15/35 | 1/12 |
| Serological diagnosis positive, n/N |  |  |  |
| Recent | 3/7 | 15/37 | 1/13 |
| Exposed | 3/7 | 13/37 | 7/13 |
| Negative | 1/7 | 9/37 | 5/13 |
| ***Mycoplasma pneumoniae*** |  |  |  |
| *Anti–M. pneumoniae* IgM in serum positive, n/N | 4/6 | 7/35 | 1/12 |
| **Cytomegalovirus** |  |  |  |
| Anti–CMV IgM in serum positive, n/N | 5/6 | 10/36 | 1/13 |
| CMV avidity IgG in serum, n/N ^c^ | 5/5 | 9/9 | 2/2 |
| Serological diagnosis |  |  |  |
| Reactivation | 5/7 | 8/36 | 1/13 |
| Negative | 2/7 | 28/36 | 12/13 |
| **Epstein Barr virus** |  |  |  |
| **anti–VCA in serum positive, n/N** | **0/7** | **3/37** | **0/13** |
| **IgG EBNA–1 in serum positive, n/N ^d^** | **ND** | **2/3** | **ND** |
| **Serological diagnosis, n/N** |  |  |  |
| Primoinfection | 0/6 | 1/37 | 0/13 |
| Reactivation | 0/6 | 2/37 | 0/13 |
| Negative | 6/6 | 34/37 | 13/13 |
| **Varicella Zoster virus** |  |  |  |
| IgM Serum positive, n/N | 0/7 | 5/35 | 0/13 |
| IgG avidity test Serum positive, n/N ^e^ | ND | 7/7* | 0 |
| Serological diagnosis, n/N |  |  |  |
| Reactivation | 0/7 | 7/37 | 0/13 |
| Negative | 7/7 | 30/37 | 13/13 |
| **Hepatitis E virus** |  |  |  |
| IgM in serum positive, n/N | 0/7 | 3/36 | 0/13 |
| **Summary of infections, n/N** |  |  |  |
| 0 | 1/7 | 5/37 | 4/13 |
| 1 | 1/7 | 21/37 | 6/13 |
| 2 or more | 5/7 | 11/37 | 3/13 |

*VZ avidity includes 5 positive and 2 indetermined IgM samples
