# Supplementary material for: Guillain-Barré syndrome after the Zika epidemic in Colombia: A multicenter, matched case-control study
Source: PLoS Negl Trop Dis. 2025 Mar 5;19(3):e0012898. doi: 10.1371/journal.pntd.0012898 (PMC11922255; doi:10.1371/journal.pntd.0012898)
Supplement: S4 Table — (DOCX) [file pntd.0012898.s004.docx]

# **S4 Table. Microbiological Tests Results by GBS Phenotype**

| **Test performed** | **Primary Demyelinating^a^**  **%** | **Primary axonal ^a^**  **%** | **Miller–Fisher ^b^**  **%** |
| --- | --- | --- | --- |
| **Zika virus** |  |  |  |
| **RT–PCR in any fluid, n/N** | **22/24** | **15/15** | **5/5** |
| Positive | 0 | 7 | 0 |
| Negative | 100 | 93 | 100 |
| **RT–PCR in serum, n/N** | **21/24** | **15/15** | **5/5** |
| Positive | 0 | 0 | 0 |
| Negative | 100 | 100 | 100 |
| **RT–PCR in urine, n/N** | **18/24** | **10/15** | **4/5** |
| Positive | 0 | 10 | 0 |
| Negative | 100 | 90 | 100 |
| **RT–PCR in CSF, n/N** | **5/24** | **5/15** | **3/5** |
| Positive | 0 | 0 | 0 |
| Negative | 100 | 100 | 100 |
| **Dengue virus** |  |  |  |
| **RT–PCR in serum, n/N** | **22/24** | **15/15** | **5/5** |
| Negative | 100 | 100 | 100 |
| **Anti–Flavivirus IgM in serum, n/N** | **22/24** | **15/15** | **5/5** |
| Positive | 5 | 0 | 0 |
| Negative | 95 | 100 | 100 |
| **Anti–Flavivirus IgG in serum, n/N** | **22/24** | **15/15** | **5/5** |
| Positive | 27 | 13 | 40 |
| Negative | 73 | 87 | 60 |
| **Flaviviruses serological, n/N** | **22/24** | **15/15** | **5/5** |
| Recent | 4 | 0 | 0 |
| Exposed | 23 | 13 | 40 |
| Negative | 73 | 87 | 60 |
| **Chikungunya virus** |  |  |  |
| **RT–PCR in serum, n/N** | **22/24** | **15/15** | **5/5** |
| Negative | 100 | 100 | 5100 |
| **RT–PCR in CSF, n/N** | **4/24** | **5/15** | **3/5** |
| Negative | 100 | 100 | 100 |
| **CHIK RT–PCR in urine, n/N** | **16/24** | **10/15** | **4/5** |
| Negative | 100 | 100 | 100 |
| **Anti–CHIK IgM in serum, n/N** | **22/24** | **14/15** | **5/5** |
| Positive | 5 | 0 | 0 |
| Negative | 95 | 100 | 100 |
| **Anti–CHIK IgG in serum, n/N** | **22/24** | **15/15** | **5/5** |
| Positive | 32 | 13 | 40 |
| Negative | 68 | 87 | 60 |
| **Serological diagnosis, n/N** | **22/24** | **14/15** | **5/5** |
| Recent | 5 | 0 | 0 |
| Exposed | 27 | 7 | 40 |
| Negative | 68 | 93 | 60 |
| ***Campylobacter jejuni*** |  |  |  |
| **Anti–*C. jejuni* IgM in serum, n/N** | **22/24** | **14/15** | **5/5** |
| Positive | 14 | 7 | 20 |
| Negative | 86 | 93 | 80 |
| **Anti–*C. jejuni* IgG in serum, n/N** | **21/24** | **15/15** | **5/5** |
| Positive | 67 | 67 | 40 |
| Negative | 33 | 33 | 60 |
| **Anti–*C. jejuni* IgA in serum, n/N** | **21/24** | **14/15** | **4/5** |
| Positive | 33 | 36 | 75 |
| Negative | 67 | 64 | 25 |
| **Serological diagnosis, n/N** | **22/24** | **15/15** | **5/5** |
| Recent | 23 | 40 | 40 |
| Exposed | 54 | 27 | 20 |
| Negative | 23 | 33 | 40 |
| ***Mycoplasma pneumoniae*** |  |  |  |
| ***Anti–M. pneumoniae* IgM in serum, n/N** | **22/24** | **15/15** | **5/5** |
| Positive | 23 | 20 | 20 |
| Negative | 64 | 73 | 80 |
| Indeterminate | 13 | 7 | 0 |
| **Serological diagnosis, n/N** | **19/24** | **14/15** | **5/5** |
| Recent | 26 | 21 | 20 |
| Negative | 74 | 79 | 80 |
| **Cytomegalovirus** |  |  |  |
| **Anti–CMV IgM in serum, n/N** | **21/24** | **15/15** | **5/5** |
| Positive | 38 | 20 | 40 |
| Negative | 62 | 60 | 60 |
| Indetermine | 0 | 20 | 0 |
| **CMV avidity IgG in serum, n/N ^c^** | **9/9** | **6/6** | **2/2** |
| Positive | 89 | 100 | 50 |
| indeterminate | 11 | 0 | 50 |
| **Serological diagnosis** | **22/24** | **15/15** | **5/5** |
| Reactivation | 36 | 60 | 20 |
| Negative | 64 | 40 | 80 |
| **Epstein Barr virus** |  |  |  |
| **anti–VCA in serum, n/N** | **22/24** | **15/15** | **5/5** |
| Positive | 9 | 0 | 0 |
| Negative | 91 | 100 | 100 |
| **IgG EBNA–1 in serum, n/N ^d^** | **2/2** | **NA** | **NA** |
| Positive | 50 | – | – |
| Negative | 50 | – | – |
| **Serological diagnosis, n/N** | **21/24** | **15/15** | **4/5** |
| Primoinfection | 5 | 0 | 0 |
| Reactivation | 5 | 0 | 0 |
| Negative | 90 | 100 | 100 |
| **Varicella Zoster virus** |  |  |  |
| **IgM Serum, n/N** | **21/24** | **15/15** | **5/5** |
| Positive | 9 | 13 | 0 |
| Negative | 91 | 87 | 100 |
| **IgG avidity test Serum, n/N ^e^** | **2/2** | **2/2** | **NA** |
| Positive | 100 | 100 | – |
| Indetermined | 0 | 0 | – |
| **Serological diagnosis, n/N** | **21/24** | **15/15** | **5/5** |
| Reactivation | 9 | 13 | 0 |
| Negative | 91 | 87 | 100 |
| **Hepatitis E virus** |  |  |  |
| **IgM in serum, n/N** | **18/24** | **15/15** | **5/5** |
| Positive | 6 | 0 | 0 |
| Negative | 94 | 100 | 100 |
| **Summary of infections, n/N** | **24/24** | **15/15** | **5/5** |
| 0 | 21 | 20 | 40 |
| 1 | 50 | 33 | 40 |
| 2 or more | 29 | 47 | 20 |

n/N indicates the number of samples processed of the total included.

^a^ Diagnosis of prymary demyelinating and primary axonal was based on neurophysiological testing of GBS cases.

^b^ Diagnosis of MFS was based on clinical profile and established criteria for GBS cases.

^c^ Testing of CMV IgG and IgG avidity test was done only when IgM was positive or equivocal to determine primoinfection (IgG avidity test negative while IgM positive) or reactivation (IgG avidity and IgM tests positive).

^d^ Testing of EBV primoinfection of reactivation was assessed only in cases with positive or equivocal anti–EBV VCA IgM antibodies using anti–EBNA IgG testing. Positive anti–EBV VCA IgM and negative anti–EBNA IgG cases were considered primoinfection, while both positive tests were considered reactivation.

^e^ Testing of VZV infection used IgG avidity assay to determine primoinfection. Only cases that were IgM–positive were tested. If the IgG OD value reduction was below 40% after urea treatment, it was considered a primary infection, if it was high (IgG OD value above 60% after urea treatment), it was considered reactivation.
