# Supplementary material for: Guillain-Barré syndrome after the Zika epidemic in Colombia: A multicenter, matched case-control study
Source: PLoS Negl Trop Dis. 2025 Mar 5;19(3):e0012898. doi: 10.1371/journal.pntd.0012898 (PMC11922255; doi:10.1371/journal.pntd.0012898)
Supplement: S5 Table — (DOCX) [file pntd.0012898.s005.docx]

# **S5 Table. Association of Microbiological Tests Results with GBS Phenotype**

| **Test performed** | **Primary Axonal ^a^**  **n** | **Primary demyelinating^a^**  **n** | **OR** | **95% CI** |
| --- | --- | --- | --- | --- |
| **Zika virus** |  |  |  |  |
| **RT–PCR in any fluid** | **15** | **22** |  |  |
| Positive | 1 | 0 | - |  |
| Negative | 14 | 22 |  |  |
| **Flavivirus Serological diagnosis** | **15** | **21** |  |  |
| Recent | 0 | 1 | **-** |  |
| Exposed | 2 | 5 | 0.5 | 0.07-3.1 |
| Negative | 13 | 16 | 1 |  |
| **Chikungunya serological diagnosis** | **14** | **22** |  |  |
| Recent | 0 | 1 | - |  |
| Exposed | 1 | 6 | 0.19 | 0.01-2.03 |
| Negative | 13 | 15 |  |  |
| ***Campylobacter jejuni* serological diagnosis** | **15** | **22** |  |  |
| Recent | 6 | 5 | 1.2 | 0.20-6.97 |
| Exposed | 4 | 12 | 0.33 | 0.05-1.95 |
| Negative | 5 | 5 | 1 |  |
| ***Mycoplasma pneumoniae serological diagnosis*** | **14** | **19** |  |  |
| Recent | 3 | 5 | 0.76 | 0.14-4.02 |
| Negative | 11 | 14 | 1 |  |
| **Cytomegalovirus serological diagnosis** | **15** | **22** |  |  |
| Reactivation | 5 | 7 | 1.07 | 0.26-4.42 |
| Negative | 10 | 15 | 1 |  |
| **Epstein Barr virus serological diagnosis** | **15** | **21** |  |  |
| Primoinfection | 0 | 1 | - |  |
| Reactivation | 0 | 1 | - |  |
| Negative | 15 | 19 | 1 |  |
| **Varicella Zoster virus serological diagnosis** | **15** | **21** |  |  |
| Reactivation | 2 | 2 | 1.46 | 0.17-12.13 |
| Negative | 13 | 19 | 1 |  |
| **Hepatitis E virus IgM in serum** | **15** | **18** |  |  |
| Positive | 0 | 1 | - |  |
| Negative | 15 | 17 | 1 |  |
| **Summary of infections** | **15** | **24** |  |  |
| 0 | 3 | 5 | 1 |  |
| 1 | 5 | 12 | 0.69 | 0.11-4.26 |
| 2 or more | 7 | 7 | 1.66 | 0.26-10.37 |
