# Supplementary material for: Guillain-Barré syndrome after the Zika epidemic in Colombia: A multicenter, matched case-control study
Source: PLoS Negl Trop Dis. 2025 Mar 5;19(3):e0012898. doi: 10.1371/journal.pntd.0012898 (PMC11922255; doi:10.1371/journal.pntd.0012898)
Supplement: S6 Table — (DOCX) [file pntd.0012898.s006.docx]

# **S6 Table. Anti-glycolipid IgG Frequency Based on Microbiological Testing and Infectious Disease Diagnosis**

| **Anti-glycolipid IgG** | **GBS cases**  **N=57 (%)** | **Controls** | | ***C. jejuni* positive** | | ***C. jejuni* negative** | | ***M. pneumoniae* positive** | | **Cytomegalovirus positive** | | **Negative to all tests performed** | |
| --- | --- | --- | --- | --- | --- | --- | --- | --- | --- | --- | --- | --- | --- |
|  |  | **Hospital**  **N=22 (%)** | **House**  **N=55 (%)** | **GBS**  **N=42 (%)** | **controls**  **N=41 (%)** | **GBS**  **N=15 (%)** | **controls**  **N=36 (%)** | **GBS**  **N=12 (%)** | **controls**  **N=11 (%)** | **GBS**  **N=16 (%)** | **controls**  **N=22 (%)** | **GBS**  **N=10 (%)** | **controls**  **N=23 (%)** |
| GM1 | 18 (32) | 1 (5) | 2 (4) | 17 (41) | 2 (5) | 1 (7) | 1 (3) | 3 (25) | 0 | 5 (31) | 2 (9) | 0 | 1 (4) |
| GM2 | 5 (9) | 1 (5) | 2 (4) | 4 (10) | 2 (5) | 1 (7) | 1 (3) | 1 (8) | 0 | 1 (6) | 0 | 0 | 1 (4) |
| GM4 | 0 | 0 | 2 (4) | 0 | 1 (2) | 0 | 1 (3) | 0 | 1 (9) | 0 | 2 (9) | 0 | 0 |
| GT1a | 12 (21) | 0 | 2 (4) | 11 (26) | 2 (5) | 1 (7) | 0 | 5 (42) | 0 | 4 (25) | 0 | 1 (10) | 0 |
| GT1b | 6 (11) | 1 (5) | 2 (4) | 4 (10) | 3 (7) | 2 (13) | 0 | 1 (8) | 0 | 1 (6) | 1 (5) | 2 (20) | 0 |
| GD1a | 7 (12) | 0 | 2 (4) | 6 (14) | 1 (2) | 1 (7) | 1 (3) | 1 (8) | 0 | 1 (6) | 0 | 1 (10) | 1 (4) |
| GD1b | 7 (12) | 0 | 2 (4) | 5 (12) | 2 (5) | 2 (13) | 0 | 1 (8) | 0 | 2 (13) | 0 | 1 (10) | 0 |
| GD3 | 10 (18) | 4 (18) | 2 (4) | 8 (19) | 5 (12) | 2 (13) | 1 (3) | 3 (25) | 0 | 4 (25) | 3 (14) | 1 (10) | 0 |
| GalC | 11 (19) | 2 (9) | 2 (4) | 10 (24) | 3 (7) | 1 (7) | 1 (3) | 2 (17) | 0 | 4 (25) | 2 (9) | 1 (10) | 0 |
| Sulfatide | 8 (14) | 1 (5) | 2 (4) | 7 (17) | 2 (5) | 1 (7) | 1 (3) | 3 (25) | 0 | 2 (13) | 1 (5) | 1 (10) | 1 (4) |
| GA1 | 5 (9) | 1 (5) | 2 (4) | 3 (7) | 2 (5) | 2 (13) | 1 (3) | 3 (25) | 0 | 3 (19) | 0 | 1 (10) | 0 |
| GQ1b | 7 (12) | 1 (5) | 2 (4) | 6 (14) | 3 (7) | 1 (7) | 0 | 1 (8) | 0 | 2 (13) | 2 (9) | 1 (10) | 0 |
| PS | 3 (4) | 2 (9) | 2 (4) | 2 (5) | 2 (5) | 1 (7) | 2 (3) | 1 (8) | 0 | 1 (6) | 1 (5) | 1 (10) | 2 (9) |
| SGPG | 2 (4) | 1 (5) | 2 (4) | 2 (5) | 2 (5) | 0 | 1 (3) | 1 (8) | 0 | 1 (6) | 2 (9) | 0 | 0 |
| LM1 | 2 (4) | 0 | 2 (4) | 2 (5) | 2 (5) | 0 | 0 | 1 (8) | 0 | 1 (6) | 0 | 0 | 0 |
| GM1:GD1a | 24 (42) | 0 | 2 (4) | 23 (55) | 3 (7) | 1 (7) | 0 | 4 (33) | 1 (9) | 6 (38) | 0 | 0 | 0 |
| GM1:GT1a | 26 (46) | 0 | 2 (4) | 25 (60) | 2 (5) | 1 (7) | 0 | 7 (58) | 0 | 8 (50) | 1 (5) | 0 | 0 |
| GM1:GQ1b | 27 (47) | 0 | 2 (4) | 24 (57) | 3 (7) | 3 (20) | 1 (3) | 6 (50) | 0 | 8 (50) | 3 (14) | 2 (20) | 0 |
| GM1:GD3 | 23 (40) | 4 (18) | 2 (4) | 21 (50) | 5 (12) | 2 (13) | 1 (3) | 5 (42) | 0 | 7 (44) | 2 (9) | 1 (10) | 1 (4) |
| GM1:Sulfatide | 23 (40) | 1 (5) | 2 (4) | 21 (50) | 3 (7) | 2 (13) | 0 | 4 (33) | 0 | 6 (38) | 1 (5) | 1 (10) | 0 |
| GD1a:GT1a | 19 (33) | 1 (5) | 2 (4) | 17 (41) | 2 (5) | 2 (13) | 1 (3) | 4 (33) | 1 (9) | 3 (19) | 0 | 2 (20) | 1 (4) |
| GD1b:GT1a | 17 (30) | 0 | 2 (4) | 16 (38) | 2 (5) | 1 (7) | 0 | 6 (50) | 0 | 6 (38) | 1 (5) | 0 | 0 |
| GalNAc–GD1a | 9 (16) | 0 | 2 (4) | 9 (21) | 2 (5) | 0 | 0 | 0 | 0 | 2 (13) | 0 | 0 | 0 |
