# Supplementary material for: Guillain-Barré syndrome after the Zika epidemic in Colombia: A multicenter, matched case-control study
Source: PLoS Negl Trop Dis. 2025 Mar 5;19(3):e0012898. doi: 10.1371/journal.pntd.0012898 (PMC11922255; doi:10.1371/journal.pntd.0012898)
Supplement: S7 Table — (DOCX) [file pntd.0012898.s007.docx]

# **S7 Table. Frequencies of Anti-glycolipid IgGs by Study Group and Conditional Logistic Models of Their Association with GBS in C. jejuni Positives in Serum**

| **Anti-glycolipid IgG** | ***C. jejuni* positive** | | **GBS cases Vs. Controls in *C. jejuni* positives** | | | |
| --- | --- | --- | --- | --- | --- | --- |
|  | **GBS**  **n=42** | **controls**  **n=41** | **OR (95% CI)** | **p–value** | **aOR (95% CI)** | **p–value** |
| GM1 | 17 (41) | 2 (5) | 12 (1·6–92·3) | 0·01 | 25^a^ (1·6–398·4) | 0·02 |
| GT1a | 11 (26) | 2 (5) | 6 (0·7–49·8) | 0·09 |  |  |
| GM1:PS | 19 (45) | 3 (7) | 14·9 (1·9–113·5) | 0·009 | 16·5^b^ (1·4–188·2) | 0·02 |
| GM1:GT1a | 25 (60) | 2 (5) | 16·9 (2·2–127·7) | 0·006 | 15·7^c^ (1·9–129·7) | 0·01 |
| GM1:GD3 | 21 (50) | 5 (12) | 14·5 (1·9–110) | 0·01 | 14·3^b^ (1·3–152) | 0·02 |
| GM1:Sulfatide | 21 (50) | 3 (7) | 15·8 (2·1–120·6) | 0·007 | 20^b^ (1·6–253) | 0·02 |
| PS:GD1a | 10 (24) | 3 (7) | 6·8 (0·8–56·8) | 0·07 |  |  |
| GD1a:GT1a | 17 (41) | 2 (5) | 12 (1·6–92·3) | 0·01 | 19·7^b^ (1·6–235·9) | 0·01 |
| GD1b:GT1a | 16 (38) | 2 (5) | 9 (1·1–71) | 0·03 | 14·7^b^ (0·7–304·5) | 0·08 |

^a^ Adjusted for sex and number of infections.

^b^ Adjusted for sex and upper respiratory infection or influenza 4 weeks before onset.

^c^ Adjusted for sex.
