# Supplementary material for: Guillain-Barré syndrome after the Zika epidemic in Colombia: A multicenter, matched case-control study
Source: PLoS Negl Trop Dis. 2025 Mar 5;19(3):e0012898. doi: 10.1371/journal.pntd.0012898 (PMC11922255; doi:10.1371/journal.pntd.0012898)
Supplement: S8 Table — (DOCX) [file pntd.0012898.s008.docx]

**S8 Table. Anti-glycolipid IgG frequency by GBS phenotype**

| **Anti-glycolipid IgG** | **GBS cases phenotype ^a^** | |
| --- | --- | --- |
|  | **Primary demyelinating**  **n=18 (%)** | **Primary axonal**  **n=15 (%)** |
| GM1 | 7 (39) | 5 (33) |
| GM2 | 3 (17) | 0 |
| PS | 1 (7) | 1 (7) |
| GM4 | 0 | 0 |
| GA1 | 3 (17) | 1 (7) |
| GD1a | 3 (17) | 1 (7) |
| GD1b | 4 (22) | 1 (7) |
| GT1a | 3 (17) | 3 (20) |
| GT1b | 2 (11) | 3 (20) |
| GQ1b | 5 (28) | 0 |
| GD3 | 4 (22) | 3 (20) |
| SGPG | 0 | 1 (7) |
| LM1 | 1 (7) | 1 (7) |
| GalNAc–GD1a | 3 (17) | 2 (13) |
| GalC | 7 (39) | 1 (7) |
| Sulfatide | 4 (22) | 1 (7) |
| GM1:GD1a | 7 (39) | 8 (53) |
| GM1:GT1a | 7 (39) | 9 (60) |
| GM1:GQ1b | 9 (50) | 10 (67) |
| GM1:GD3 | 6 (33) | 9 (60) |
| GM1:Sulfatide | 7 (39) | 8 (53) |
| GD1a:GT1a | 6 (33) | 3 (20) |
| GD1b:GT1a | 5 (28) | 4 (27) |

^a^ Matched GBS cases with neurophysiology consistent with primary demyelinating primary axonal.
