# Supplementary material for: Guillain-Barré syndrome after the Zika epidemic in Colombia: A multicenter, matched case-control study
Source: PLoS Negl Trop Dis. 2025 Mar 5;19(3):e0012898. doi: 10.1371/journal.pntd.0012898 (PMC11922255; doi:10.1371/journal.pntd.0012898)
Supplement: S1 Appendix — (DOCX) [file pntd.0012898.s009.docx]

Additional members of NEAS Network

| Name | City | Affiliation |
| --- | --- | --- |
| Guillermo González-Manrique | Neiva | Hospital Universitario de Neiva Hernando Moncaleano Perdomo, Neiva, Colombia |
| Jorge Alberto Angarita | Neiva | Clínica Medilaser, Neiva, Colombia. |
| Paula Barreras | USA | Departments of Neurology, Johns Hopkins University School of Medicine, Baltimore, MD, USA |
| Cyndi Lorena Beltran | Neiva | Hospital Universitario de Neiva Hernando Moncaleano Perdomo, Neiva, Colombia |
| Laura Sofia Muñoz | USA | Departments of Neurology, Johns Hopkins University School of Medicine, Baltimore, MD, USA |
| Luis Carlos Quintero-Malo | Neiva | Clínica Medilaser, Neiva, Colombia. |
| Katherine V. Claros | Neiva | Hospital Universitario de Neiva Hernando Moncaleano Perdomo, Neiva, Colombia |
| Angela Catalina Vallejo-Cajiga | Pasto | Hospital Universitario Departamental de Nariño, Pasto, Colombia |
| Liliana Montenegro | Pasto | Hospital Universitario Departamental de Nariño, Pasto, Colombia |
| William Jaramillo | Pasto | Hospital Universitario Departamental de Nariño, Pasto, Colombia |
| Thanya Lagos | Pasto | Hospital Universitario Departamental de Nariño, Pasto, Colombia |
| Maria A. Garcia-Dominguez | USA | Departments of Neurology, Johns Hopkins University School of Medicine, Baltimore, MD, USA |
| Maria F. Ramos-Sanchez | Cúcuta | Hospital Universitario Erasmo Meoz, Cúcuta, Colombia |
| Nelson Rivera-Franco | Cali | Department of Microbiology, Universidad del Valle, Cali, Colombia |
| Andrés Castillo | Cali | Department of Biology, Universidad del Valle, Cali, Colombia |
| Diana López-Álvarez | Cali | Department of Microbiology, Universidad del Valle, Cali, Colombia |
| José Miguel Enciso-Gutierrez | Cali | Department of Microbiology, Universidad del Valle, Cali, Colombia |
| Federico Arturo Silva-Sieger | Bucaramanga | Hospital Internacional de Colombia, Bucaramanga, Colombia |
| Jenny P. Garzón | Bucaramanga | Hospital Internacional de Colombia, Bucaramanga, Colombia |
| Daniela Noguera | Barranquilla | La Misericordia Clínica Internacional, Barranquilla, Colombia |
| Diego F. Lizarazo-Ortega | Cucuta | Hospital Universitario Erasmo Meoz, Cúcuta, Colombia |
| Melissa M. Gomez-Arrieta | Bucaramanga | Hospital Internacional de Colombia, Bucaramanga, Colombia |
| José Rafael Tovar-Cuevas | Cali | School of Statistics, Universidad del Valle, Cali, Colombia |
| Laura L. Domínguez-Barrios | Cali | School of Statistics, Universidad del Valle, Cali, Colombia |
| Andres Mauricio Gonzalez-Rios | Cali | Department of Microbiology, Universidad del Valle, Cali, Colombia |
| Eder de Jesús Mosquera-Vivas | Cali | School of Public Health, Universidad del Valle, Cali, Colombia |
| Lina María Chaucanés | Cali | School of Public Health, Universidad del Valle, Cali, Colombia |
| Erica Aristizábal | Cali | Department of Microbiology, Universidad del Valle, Cali, Colombia |
| Maria Camila Gil-Avendaño | Cali | Department of Microbiology, Universidad del Valle, Cali, Colombia |
