# Supplementary material for: Guillain-Barré syndrome after the Zika epidemic in Colombia: A multicenter, matched case-control study
Source: PLoS Negl Trop Dis. 2025 Mar 5;19(3):e0012898. doi: 10.1371/journal.pntd.0012898 (PMC11922255; doi:10.1371/journal.pntd.0012898)
Supplement: Attachment — Submitted filename: Reply to reviewers comments.docx [file pntd.0012898.s011.docx]

**Response to the Reviewers**

We sincerely appreciate the reviewers’ positive reviews of the manuscript and the valuable suggestions to enhance the clarity of the data and results. The reviewers’ recommendations have been extremely useful in guiding our revisions. Accordingly, we have made the suggested changes to the manuscript, which we believe have significantly improved its quality. This document provides our detailed responses to each comment and explains the specific revisions made.

Reviewer #1:

1. Comment: *The objectives of the study should be clearly described in the abstract.*

R/ The objective of the study was added in the abstract. “The present study assessed the role of preceding infections, including arboviruses and other pathogens, as risk factors for GBS*”*

2. Comment: *The authors should mention whether the house controls were age-sex matched with the cases.*

R/ House controls were not age-sex matched with the cases. This was added to the methods

3. Comment: *In Table 1, % of patients with more than 15 years were presented. It would be better, if the data of different age categories could be presented. Also it would be interesting to see the infection profiles in different age groups e.g. childhood GBS, young adults or elderly and give a comparison. Authors are requested to perform a sub-analysis if possible.*

R/ The categorization at 15 years old was decided on pediatric and non-pediatric GBS cases. We followed the recommendation, and the suggested sub-analysis by age group was added in the text of the results section, in Table 2 and supplemental Table 3.

4. Comment: *Is there any data of infection profile before the Zika epidemic in Colombia? If yes, it would be interesting to compare the pre and post Zika period findings in the discussion section.*

R/Unfortunately, we were unable to find information on infection profile before the Zika epidemic in Colombia.

5. Comment: *Some abbreviations are used inside the text. The full form of the abbreviations should be mentioned where they are first used.*

R/ the entire document was reviewed to ensure abbreviations are described in full where first used.

Reviewer #2:

Comment: *The study provides a clear and comprehensive presentation of the data, with a detailed analysis of the clinical, microbiological, and neurological characteristics of patients with Guillain-Barré Syndrome (GBS) and controls. The use of standardized approaches for biological sample collection and clinical and neurophysiological assessment strengthens the validity of the results. The study also benefits from a multicentric approach covering various ecological regions in Colombia.*

R/ We thank the reviewer for these comments

Reviewer #3:

1. Comment: Why is the Modified Rankin Scale (MRS) being used and not Hughes GBS Disability Score (HGDS) for the disability assessment? Conventionally, MRS is used for stroke, and HGDS is used for GBS.

R/ We greatly appreciate the reviewer for this comment. Both scales are very valuable as outcome assessments in GBS and share similarities. We have now included the HGDS in the methods for outcome evaluation and reported the results and Table 2.

2. Comment: *The authors used Hadden criteria to subclassifying the electrodiagnostic subtypes, but I did not see the EDx subtypes in the results. Which EDx subtype is more commonly associated with certain pathogen? For example, axonal or demyelinating is more commonly associated with C. jejuni or CMV or etc? And what is the association of the EDx subtypes with the anti-glycolipid antibodies?*

R/ The EDx as related to microbiological results are included in the results and supplemental Table 4 “Cases of pure motor showed a high frequency of CMV reactivation (60%) as compared with sensorimotor (36%) and MFS variants (20%). Similarly, cases of pure motor had a high frequency of 2 or more co-infections (47%) as compared with sensorimotor (29%) and MFS (20%) (Supplemental Table 4).” The EDx subtypes in relation to anti-glycolipid antibodies are included in the results and supplemental table 7 “Although there was a higher frequency of anti-heteromeric complexes IgG antibodies among pure motor cases, a relative high frequency of such antibodies was also observed in sensorimotor cases (supplemental table 7)”

3. Comment: *Diarrhea as the presenting symptom is significant here, but not diarrhea 4 weeks before onset – Do you mean diarrhea as the presenting symptom of GBS? We are more interested in diarrhea as the antecedent illness, rather than the presenting symptoms here. Could you clarify?*

R/ Both Diarrhea as presenting symptom and antecedent were collected and analyzed. Both were more frequent among cases than controls. However, one person among controls reported diarrhea as an antecedent, which could affect statistical power and explain the lack of statistical association between diarrhea as a preceding illness and GBS. This has been added to the discussion.
