# Supplementary material for: Guillain-Barré syndrome after the Zika epidemic in Colombia: A multicenter, matched case-control study
Source: PLoS Negl Trop Dis. 2025 Mar 5;19(3):e0012898. doi: 10.1371/journal.pntd.0012898 (PMC11922255; doi:10.1371/journal.pntd.0012898)
Supplement: Attachment — Submitted filename: Response letter PLOS NTD 1_19_2025.docx [file pntd.0012898.s012.docx]

Reviewer #3: 
*1. The authors seem do not understand the difference between diarrhea as presenting symptom or as preceding illness/symptom. This is rather confusing, as the main objective of the study is to determine the role of preceding illness in triggering GBS. It is very hard to imagine diarrhea as the presenting symptoms in GBS, as the autonomic features would not have occurred in the early phase, when it is present, it should cast doubt on the diagnosis.*

R/ We appreciate the reviewer’s comment and understand the distinction made. In our manuscript, we specifically differentiated between presenting symptoms -clinical features observed at the moment of the onset of neurological symptoms- and preceding symptoms, which occur prior to the onset of neurological symptoms. Several published GBS studies, including case-control studies, have considered preceding diarrhea within 4-6 weeks before GBS onset, often without clarifying whether diarrhea was present at neurological symptom onset. We believe it is important to distinguish between the concept of preceding diarrhea and diarrhea observed at the time of GBS presentation, especially in tropical regions where *Campylobacter jejuni*, a pathogen implicated in GBS pathogenesis, is frequently associated with diarrhea lasting for several days. Diarrhea may also occur in certain viral infections that can trigger GBS. Importantly, we do not interpret diarrhea present at the onset of GBS symptoms as a sign of autonomic dysfunction. Furthermore, diarrhea is not a symptom that disqualifies the diagnosis of GBS as it is recognized by the guidance for diagnosis of GBS as outlined recently by the European Academy of Neurology/Peripheral Nerve Society Guideline on diagnosis and treatment of Guillain–Barré syndrome (van Doorn et. al. *Eur J Neurol*. 2023;30:3646–3674). We introduced changes in table 2 to further clarify the distinction between presenting and preceding diarrhea.

*2. The authors also do not seem to understand the electrodiagnostic subtype of the GBS when the Hadden criteria were used. It is imperative to know whether the axonal or the demyelinating subtypes were more commonly associated with certain pathogens and anti-glycolipids.*

*R/* We appreciate the reviewer’s comment as we understand the distinction established by the electrophysiological criteria described by Hadden et. al. in 1998. In our study, we adhered to this classification while employing the terminology recently recommended by the European Academy of Neurology/Peripheral Nerve Society Guideline on diagnosis and treatment of Guillain–Barré syndrome (van Doorn et. al. *Eur J Neurol*. 2023;30:3646–3674). Specifically, the terms “primary demyelinating” and “primary axonal” described by Hadden closely correspond to the motor sensory and motor subtypes. We agree with the reviewer to adhere to the Hadden’s criteria terminology and we made the corresponding changes of terms in the text and tables. Supplementary Table 4 provides a comparison between GBS subtypes and microbiological findings. In earlier versions of the manuscript, we did not conduct statistical comparisons for GBS subtypes due to the small sample size, which limits statistical power. Additionally, we had already performed multiple comparisons in the primary analysis and corrected for them, and further subgroup analyses risk increasing the likelihood of spurious associations. As requested by the reviewer, we have now included a new supplementary table (sTable 5) presenting the odds ratios (OR) and their corresponding 95% confidence intervals. As expected, no statistically significant associations were found. The ORs are now included in the Results section. We provided the descriptive comparison in the updated Supplementary Table 8 (previously *Table 7*) regarding the association between GBS subtypes and anti-glycolipid antibodies. However, given the limited sample size, no statistical tests were conducted for the abovementioned reasons.

*3. Lastly, the inclusion criteria of the hospital controls are also doubtful in this study. In the Methods, it was stated that patients admitted with febrile illness or suspected acute viral infection were included as hospital controls. How would one know whether this cohort of patients may develop the GBS later? The more appropriate hospital controls would be patients that were admitted for other neurological diseases (other than GBS) after the febrile illness/viral infection.*

R/ We appreciate this comment, as it allows us to further clarify the study design regarding the selection of control groups. One of the control groups consisted of hospital controls, defined as patients presenting with acute febrile illnesses but without clinical or diagnostic evidence of neurological involvement. These controls were specifically selected to reflect the hypothesized exposure (infections) without the outcome of interest (GBS). This approach aligns with the fundamental principle of case-control studies: “The purpose of the control group is to provide information about the exposure distribution in that source population.” In this context, the hospital controls allow a direct testing of the hypothesis that specific infections (pathogens) are associated with GBS by ensuring the control group represents similar exposure opportunities to those in the case group. We have included this clarification in the method section.

Other neurological diseases used as controls may not provide the same exposure opportunities to infections as GBS patients. In our study, we explored which neurological conditions might serve as suitable controls for our hypothesis but could not identify a suitable list of conditions with a similar background exposure to that expected in our source population. Moreover, some infections (e.g., arbovirus, CMV) are associated with other neurological conditions, such as encephalitis, which could bias the association towards the null.

Regarding the concern that controls might later develop GBS, we emphasize that the selection of controls was concurrent with the cases (“concurrent controls”). Both cases and controls are identified during the same timeframe to ensure equal exposure opportunities to risk factors. The hypothesized infectious triggers of GBS (e.g., specific pathogens) generally act within a short timeframe (1–4 weeks). If a control were to develop GBS later, the exposure responsible for their condition would occur after their inclusion as a control, preventing bias in the current analysis. Furthermore, controls who later developed GBS would have been included as cases in the study without introducing bias.
